# Amino acids and insulin act additively to regulate components of the ubiquitin-proteasome pathway in C2C12 myotubes

**DOI:** 10.1186/1471-2199-8-23

**Published:** 2007-03-19

**Authors:** Fouzia Sadiq, David G Hazlerigg, Michael A Lomax

**Affiliations:** 1 Division of Cell & Molecular Biology, Imperial College London, Wye Campus, Wye, Ashford, Kent, TN25 5AH, UK; 2 School of Biological Sciences, University of Aberdeen, Aberdeen, AB24 5UA, UK; 3 Institute of Medical Sciences, Foresterhill, University of Aberdeen, Aberdeen, AB25 2ZD, UK

## Abstract

**Background:**

The ubiquitin-proteasome system is the predominant pathway for myofibrillar proteolysis but a previous study in C2C12 myotubes only observed alterations in lysosome-dependent proteolysis in response to complete starvation of amino acids or leucine from the media. Here, we determined the interaction between insulin and amino acids in the regulation of myotube proteolysis

**Results:**

Incubation of C2C12 myotubes with 0.2 × physiological amino acids concentration (0.2 × PC AA), relative to 1.0 × PC AA, significantly increased total proteolysis and the expression of 14-kDa E2 ubiquitin conjugating enzyme (p < 0.05). The proteasome inhibitor MG132 blocked the rise in proteolysis observed in the 0.2 × PC AA media. Addition of insulin to the medium inhibited proteolysis at both 0.2 and 1.0× PC AA and the expression of 14-kDa E2 proteins and C2 sub unit of 20 S proteasome (p < 0.05). Incubation of myotubes with increasing concentrations of leucine in the 0.2 × PC AA media inhibited proteolysis but only in the presence of insulin. Incubation of rapamycin (inhibitor of mTOR) inhibited amino acid or insulin-dependent p70 S6 kinase phosphorylation, blocked (P < 0.05) the inhibitory effects of 1.0 × PC AA on protein degradation, but did not alter the inhibitory effects of insulin or leucine

**Conclusion:**

In a C2C12 myotube model of myofibrillar protein turnover, amino acid limitation increases proteolysis in a ubiquitin-proteasome-dependent manner. Increasing amino acids or leucine alone, act additively with insulin to down regulate proteolysis and expression of components of ubiquitin-proteasome pathway. The effects of amino acids on proteolysis but not insulin and leucine, are blocked by inhibition of the mTOR signalling pathway.

## Background

Starvation induces muscle wasting by decreasing the rate of muscle protein synthesis and increasing protein degradation. A decrease in plasma concentrations of amino acids, and in particular branched-chain amino acids, is thought to synergise with lower levels of insulin to signal the starvation-induced decrease in protein synthesis [1] but the mechanisms responsible for the up regulation of muscle proteolysis are less well characterised. The major proteolytic systems that are activated in atrophying skeletal muscle are lysosomal, calcium dependent and ATP dependent ubiquitin-proteasome pathway [2]. Of these, the latter is considered the predominant biological mechanism controlling protein degradation in myofibrillar proteins in skeletal muscle [3]. This proteolytic pathway comprises of a cascade of ATP dependent enzymatic reactions in which ubiquitin protein is activated by ubiquitin activating enzyme, E1, which then transfers ubiquitin to ubiquitin conjugating enzyme, E2. This assembly then binds either directly to protein substrate or involves another ligating enzyme, E3. The cycle continues to produce a polyubiquitinated protein substrate which then enters the 26 S proteasome complex for rapid degradation. The 20 S proteasome, catalytic core of 26 S proteasome, comprises subunits having peptidase activity, which digest proteins [4]. Increased expression of 14-kDa E2 and E3 ubiquitin ligases, atrogin-1/MAFbx and MuRF1 has been observed in atrophying muscle [5,6].

Low level of plasma insulin triggers protein degradation in muscle through activation of the ubiquitin-proteasome pathway [7,8] while higher levels down regulate the expression of 14-kDa E2 conjugating enzyme proteins *in vitro *[9]. *In vivo*, a 6-hour hyperinsulinaemic, hyper-aminoacidaemic and euglycaemic clamp significantly reduced the mRNA expression for ubiquitin in fast twitch and mixed skeletal muscle [10]. Constant infusions of glucose and a mixture of essential amino acids in calves significantly attenuated mRNA expression for C2 sub unit of 20 S proteasome [11]. Observations in hepatoma cells have revealed that insulin regulates its anticatabolic activity by decreasing ubiquitin mediated proteasomal activity [12]. Despite the evidence that the ubiquitin-proteasome pathway may be responsible for the increased rates of muscle proteolysis during fasting, *in vitro *studies in C2C12 myotubes and hepatocytes have suggested that lysosome-dependent autophagic pathway is responsible for proteolytic responses to complete starvation of amino acids or from complete media [13,14].

Signalling cascades that regulate intracellular protein phosphorylation during nutrient deficiency and hormonal imbalance are not well characterised. Studies in various cell types have demonstrated that the mammalian target of rapamycin (mTOR) signalling pathway, integrates the effects of insulin and amino acids on muscle protein synthesis through phosphorylation of two key regulators of translation, 70 kDa ribosomal protein S6 kinase (p 70 S6) and eukaryotic initiation factor 4 E binding protein-1 (4EBP-1) [15,16]. mTOR has also been identified as an important protein kinase in the starvation-signalling pathway of autophagy [17] and activation of S6 kinase is associated with autophagy in rat hepatocytes [18] C2C12 myotubes [13].

In this paper we test the hypothesis that the antiproteolytic actions of insulin and amino acids in C2C12 myotubes are mediated by the ubiquitin-dependent proteasome pathway. Our data demonstrate that low levels of amino acids increase ubiquitin-proteasome-dependent proteolysis in myotubes and that amino acids act additively with insulin.

## Results

**Figure 1:**
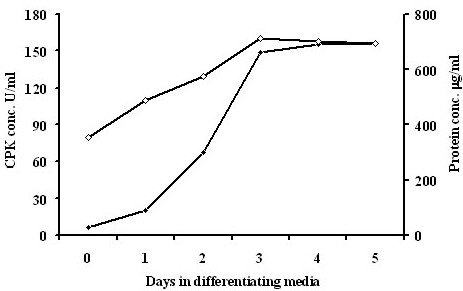
**CPK and protein measurements**. CPK (open diamonds) and protein (filled diamonds) concentration in C2C12 myotubes during 5 days incubation in differentiating media

It is an inherent characteristic of C2C12 myoblasts that they fuse into multinucleated myotubes when the level of serum in the incubation medium is reduced. The expression of many muscle specific genes such as creatine kinase, desmin and myosin heavy chain is increased during myogenic differentiation [19,20]. Differentiation of myotubes was confirmed by approximately 30-fold increase in CPK activity relative to unfused myoblasts (Figure 1.) and by visualisation under phase contrast microscopy of elongated, multinucleated cells. CPK activity remained high under all incubation conditions (results not shown).

### Effect of insulin on total protein degradation

**Figure 2:**
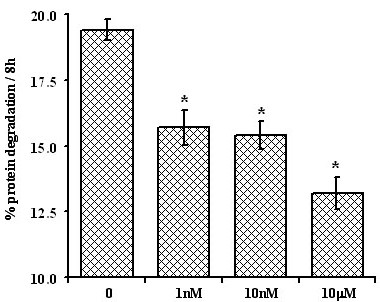
**Effect of insulin on protein degradation**. Long-lived proteins in C2C12 myotubes were labelled with 1 μCi _L_[ring -3, 5-^3^H]-tyrosine/ml fusion medium for 48 hours. Myotubes were rinsed twice with 1 × HBSS and incubated in 1 × PC AA media for 2 hours. Myotubes were rinsed and incubated with 10^-9^, 10^-8 ^and 10^-5 ^M insulin for 8 hours. Samples were collected and percent protein degradation was calculated as described in material and methods. Values are means ± SEM from 5 different experiments performed in at least triplicates. * Represents significant difference compared to control (zero insulin).

Initially we aimed to determine the concentration of insulin that can significantly inhibit proteolysis during 8 hours of incubation. Insulin inhibited the release of TCA soluble radioactivity from C2C12 myotubes pre-labelled with _L_[ring -3, 5-^3^H]-tyrosine at all of the concentrations employed with the half-maximal effect on proteolysis of approximately 10 ^-8 ^M (P < 0.01) (Figure 2).

### Amino acids and insulin act additively to regulate total proteolysis

**Figure 3:**
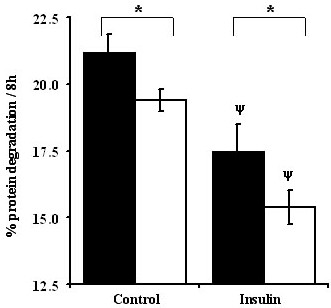
**Amino acids and insulin act additively to regulate total proteolysis in C2C12 myotubes**. Experimental procedures are the same as described under Figure 2. Values are means ± SEM for 5 different experiments performed in at least triplicates. * Indicates differences in protein degradation between 0.2 × PC AA (filled bars) & 1.0 × PC AA (open bars) treatments. ψ Represents differences between insulin versus control (without insulin) groups.

Physiological (1.0 × PC AA) and sub-physiological concentration of amino acids (0.2 × PC AA) were used to determine the interaction between amino acids and insulin in the regulation of skeletal muscle protein degradation. Protein degradation was increased by 9–12% when differentiated myotubes were incubated with the amino acid limiting 0.2 × PC AA media compared with 1.0 × PC AA, in both the absence and presence of insulin (P < 0.001; Figure 3). Insulin addition (10 ^-8 ^M) to the media decreased protein degradation (18 to 21%) at both concentrations of amino acids (p < 0.001; Figure 3). The inhibitory effects of amino acids and insulin on protein degradation in differentiated C2C12 cells were additive.

### Proteasome inhibitor MG132 attenuates amino acid mediated total proteolysis

**Figure 4:**
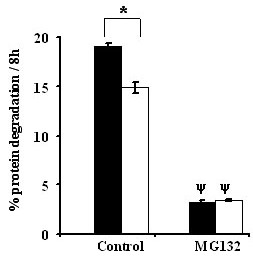
**MG132 blocks amino acid mediated total proteolysis**. Experimental procedures are the same as described under Figure 2. Values are means ± SEM for 2 different experiments performed in at least triplicates. * Indicates differences in protein degradation between 0.2 × PC AA (filled bars) & 1.0 × PC AA (open bars) treatments. ψ Represents differences between MG132 (50 uM) versus control (without MG132) groups.

Next we employed the proteasome inhibitor MG132 (50 μM) to determine the role of the 26S proteasome pathway in responses to varying amino acid concentration. MG132 inhibited proteolysis by about 75% in incubations with both physiological and sub-physiological concentrations of amino acids, but importantly, the inhibitor abolished the difference in proteolysis between the two amino acid levels (P < 0.0001; Fig 4). This result demonstrated that media amino acid depletion increased ubiquitin-proteasome-dependent-proteolysis in C2C12 myotubes.

### Amino acids and insulin down regulate expression of components of ubiquitin-proteasome pathway

**Figure 5:**
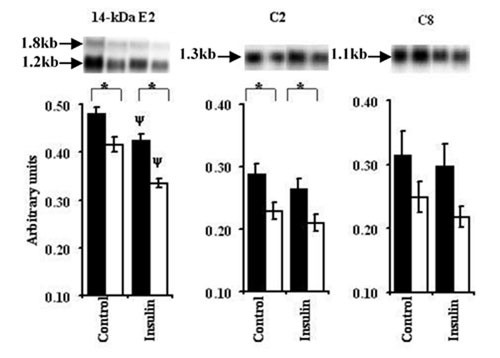
**Effect of amino acids and insulin on components of ubiquitin-proteasome pathway**. Experimental procedure is the same as described under Figure 2 except that instead of _L_[ring -3, 5-^3^H]-tyrosine cold tyrosine was used to simulate similar conditions. Values are means ± SEM for 3–4 experiments performed in triplicates. * Indicates differences in protein degradation between 0.2 × PC AA (filled bars) & 1.0 × PC AA (open bars) treatments while ψ represents differences between insulin versus control (without insulin) groups. Autoradiographic signals were normalised for GAPDH expression. In case of 14-kDa E2, autoradiographic signals for 1.2 kb transcript were normalised. Mean normalised levels of gene expression are represented as arbitrary units.

Having observed significant additive changes in total protein degradation in response to increasing amino acid and insulin media concentrations, we next sought to discover whether changes in the expression of components of the ubiquitin-proteasome pathway were also sensitive to these treatments. Compared to 1 × PC AA, the 0.2 × PC AA medium elicited a significant increase in mRNA expression of the 14-kDa E2 conjugating enzyme of the ubiquitin-proteasome pathway in C2C12 myotubes (P < 0.05; Figure 5). When insulin was added to the medium there was a decrease in the expression of the gene encoding 14-kDa E2 in the presence of both 1.0 × and 0.2 × PC AA (P < 0.001). Insulin and amino acids were observed to have additive inhibitory effects on 14-kDa E2 gene expressions.

Next we sought to determine if the C2 and C8 subunits of the 20 S proteolytic core of the proteasome were also responding to the availability of amino acids and insulin. The expression of the gene encoding C2 was reduced by 21% when C2C12 myotubes were treated with 1.0 × PC AA, compared to 0.2 × PC AA (P < 0.05). Insulin addition to media containing either the low or physiological amino acid concentrations further decreased C2 mRNA abundance in the myotubes, compared to that observed in the absence of insulin (P < 0.05). Incubation with the 1.0 × PC AA compared with 0.2 × PC AA elicited an inhibitory effect on the expression of C8 in the differentiated C2 C12 incubations but this was not statistically significant.

### Leucine increases the inhibitory effect of insulin on protein degradation

**Figure 6:**
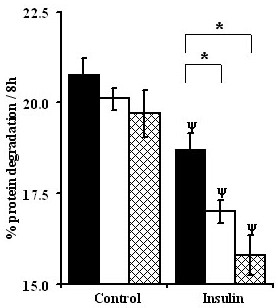
**Leucine increases the inhibitory effect of insulin on protein degradation in C2C12 myotubes**. Fully differentiated myotubes were pre labelled with _L_[ring -3, 5-^3^H]-tyrosine for 48 hours. Myotubes were rinsed twice with 1 × HBS and chased in 0.2 × PC AA medium for 2 hours followed by incubation with 30 (filled bars), 150 (open bars) and 750 (hatched bars) μM leucine in 0.2 × PC AA medium. Values are means ± SEM for two experiments performed in quadruplets. * Represents significant differences among leucine levels whereas ψ indicates differences relative to control groups.

We next assessed the specific effect of leucine on myotube protein degradation by supplementing the amino acid limiting 0.2 × PC AA media with three levels of leucine ranging from sub-physiological to supra-physiological (30 μM, 150 μM (physiological concentration) and 750 μM). In the absence of insulin, increasing the concentration of leucine in the media tended to inhibit myotube protein degradation but this effect was not significant (P > 0.05). In the presence of insulin, proteolysis was progressively inhibited by the addition of increasing leucine concentration with both 150 and 750 μM leucine, being significantly lower compared to 30 μM leucine (P < 0.001; Figure 6). These results differed from the inhibitory response observed in the absence of insulin when the concentration of the physiological mixture of amino acids was increased (Figure 3) and suggest that the effects of amino acids supply are not simply due to variations in leucine concentration. The results also indicate that the inhibitory effect of insulin on myotube protein degradation is dependent on the presence of a physiological concentration of leucine in the media.

### Leucine can modulate the expression of components of ubiquitin-proteasome pathway

**Figure 7:**
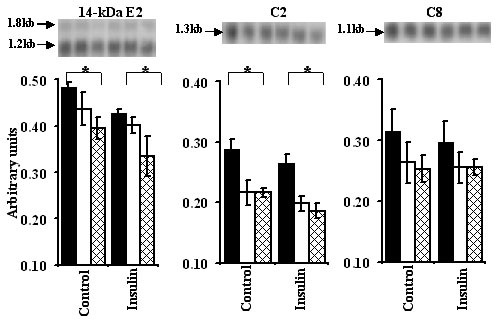
**Effect of insulin and 30 (filled bars), 150 (open bars) and 750 (hatched bars) μM leucine on ubiquitin-proteasome pathway**. Experimental procedure is the same as described under Figure 6. Autoradiographic signals normalised for GAPDH were analysed as described under fig 5. * Symbolises significant difference between 30 and 750 μM leucine.

There was a dose dependent decrease in myotube 14-kDa E2 expression in response to increasing leucine concentrations, both in the presence or absence of insulin and these effects of leucine and insulin were additive (P < 0.05; Figure 7). Compared to 30 μM, 750 μM leucine caused a significant reduction in myotube expression of C2, both in the presence and absence of insulin (P < 0.05). No significant effects of leucine on C8 expression were observed.

### Amino acids but not leucine, regulate protein degradation and 14-kDa E2 expression through the mTOR signalling pathway

**Figure 8:**
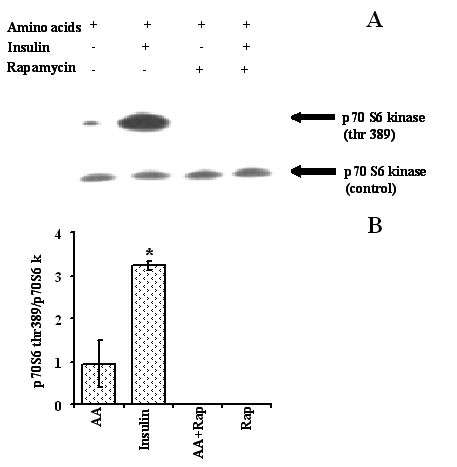
**Effect of rapamycin on p 70 S6 kinase phosphorylation status in C2C12 myotubes incubated with insulin and 1.0 × PC AA**. Myotubes were serum starved overnight and next morning treated with rapamycin (2.2 × 10^-8 ^M) in 1.0 × PC AA for 20 min. Half of the rapamycin treated or non treated cells were then stimulated with insulin (10^-8 ^M) for another 20 min. A) immunodetection on western blot, B) respective densitometric analyses. Values are mean ± SEM from one experiment performed in duplicates.

The studies reported to date have suggested that both amino acids and insulin act on protein synthesis through the mTOR signalling pathway [15]. We therefore tested the hypothesis that the effects of amino acids and insulin, on protein degradation in C2C12 myotubes are also mediated through the mTOR pathway using rapamycin, a well defined inhibitor of mTOR. In order to validate if rapamycin was inhibiting phosphorylation of p 70 S6 kinase, western blotting followed by immunodetection was performed. Rapamycin completely abolished p70 S6 kinase phosphorylation at Thr 389 site (index of total S6 activity *in vivo *[21]. Uniform bands across the treatments after reprobing the blot with total p70 S6 kinase antibody confirmed that the absence of phosphorylated p70 S6 kinase was not merely because of lack of S6 activity in the samples (Figure 8).

**Figure 9:**
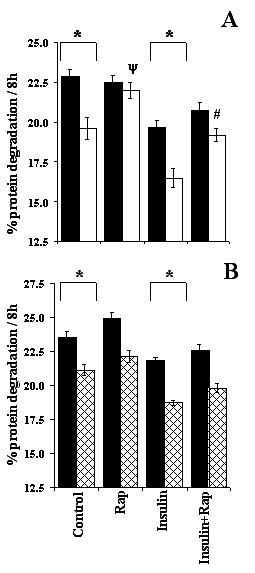
**Effect of rapamycin on total protein degradation**. Myotubes were incubated with 0.2 × PC AA (filled bars) or 1.0 × PC AA (open bars) media (A), or with 30 (filled bars) and 750 (hatched bars) μM leucine in 0.2 × PC AA medium (B). After labelling C2C12 myotubes for 48 hours, cells were incubated with 0.2 × PC AA or 1.0 × PC AA for two hours. Rapamycin (RAP) 2.2 × 10^-8 ^M was then added to half of the wells containing 0.2 × PC AA or 1.0 × PC AA and dishes transferred back to incubator. Twenty minutes later insulin 10^-8 ^M was added in to half of the rapamycin treated and half of the untreated cells and incubated at 37°C for another 8 hours. Since rapamycin has half-life of about 3–4 hours media was supplemented with rapamycin after the first four hours of incubation. Data represent mean ± SEM from one or two experiments performed in triplicates. * Indicates differences between two amino acids concentrations, ψ represents rapamycin treated versus non-rapamycin treated (control) group and # symbolises difference between insulin and rapamycin treated versus insulin treated groups.

Myotubes were challenged with rapamycin followed by stimulation with insulin. Rapamycin treatment of cells did not alter proteolysis during incubations with 0.2 × PC AA, in the presence and absence of insulin (Figure 9A). Addition of rapamycin, however, blocked the inhibitory effect on protein degradation of increasing media amino acid concentrations from 0.2 × PC AA to 1.0 × PC AA, with and without insulin (P < 0.001; Figure 9a). Rapamycin did not block the inhibitory action of insulin, on myotube protein degradation. In contrast to the effects of low concentrations of a physiological mixture of amino acids, the stimulatory effect of decreasing the media leucine concentration from 750 μM to 30 μM, on myotube protein degradation was not blocked in the presence of rapamycin (P > 0.05; Figure 9b). As in the previous experiment, rapamycin also failed to alter the inhibitory effect of insulin on protein degradation.

**Figure 10:**
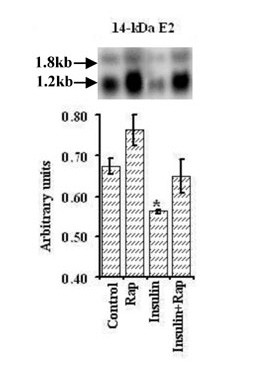
**Effect of rapamycin on the expression of 14-kDa E2 ubiquitin conjugating enzyme in C2C12 myotubes incubated with or without insulin in 1× PC AA**. The same experimental procedure was employed as described under Figure 9A. Values represent mean ± SEM from a single experiment performed in triplicates. * Symbolises significant difference relative to control group.

Rapamycin increased the expression of the ubiquitin conjugating enzyme 14-kDa E2 in both the presence and absence of insulin during incubations with 1.0 × PC AA, although these changes did not attain statistical significance (Figure 10).

Taken together these studies with rapamycin demonstrate that varying the concentration of an amino acid mixture influences proteolysis and expression of 14-kDa E2, in C2C12 myotubes, via the mTOR pathway whereas leucine and insulin act through other pathway(s).

## Discussion

The anabolic effect of insulin on muscle protein synthesis is acutely sensitive to amino acids [1]. Studies on muscle protein degradation have suggested that amino acids also influence insulin action but few studies have investigated the specific cellular pathway involved [22]. The ATP-dependent ubiquitin-proteasome pathway is the most likely target for control by insulin and amino acids since proteasome-dependent-proteolytic activity and expression of components of this pathway are stimulated by starvation and inhibited by refeeding and insulin [23]. However, a previous study of protein degradation in C2C12 myotubes could only find evidence of alterations in lysosome-dependent proteolysis in response to complete starvation of amino acids or leucine from the media [13]. We therefore carried out a study to examine the effect of more physiological alterations in amino acid concentration on the effect of insulin on the ubiquitin-proteasome pathway in C2C12 myotubes.

This study is the first to report that the amino acids alone, or additively with insulin, decrease muscle protein degradation by acting on the ubiquitin dependent proteasome pathway. We also report that varying the concentration of leucine whilst maintaining all other amino acids at a low level, does not mimic the effect of varying all amino acid concentrations suggesting that the response to amino acids is more than a response to alterations in leucine as has been previously suggested [13].

Increasing the media amino acid concentration from 0.2 × physiological levels to 1.0 × PC AA decreased myotube proteolysis as assayed by the release of protein-bound labelled tyrosine into the medium, but these effects were abolished by addition of the proteasome inhibitor MG132. Furthermore, the expression of both the 14-kDa E2 and C2 sub unit of 20 S proteasome was significantly increased during incubations with the sub-physiological amino acid incubation medium. The effect of amino acids was additive with insulin: maximal stimulation of protein degradation and expression of 14-kDa E2 and C2 were observed in the absence of insulin and at 0.2 × amino acids. Addition of insulin in the presence of amino acids strongly inhibited proteolysis and expression of 14-kDa E2 and C2. Depletion or supplementation of media leucine concentration influenced proteolysis but only in the presence of insulin. These results therefore strongly argue that insulin and amino acids interact to suppress myotube proteolysis by acting on the ubiquitin-proteasome pathway. The tyrosine release assay measures total myotube protein degradation and is therefore more representative of muscle protein degradation and is also able to determine small differences in turnover that are not possible with the measurement of specific proteins or their rate of ubiquitlylation.

A previous study in C2C12 cells [13] was unable to find any effects of amino acid or leucine starvation in the media on proteasome-dependent proteolysis or expression of components of this pathway. Our current study differed from the previous study in two ways: firstly, we examined the effect of physiological changes in amino acid concentrations by decreasing to 0.2 × physiological levels or in the case of leucine, altering the concentrations whilst maintaining the others at 0.2 × physiological levels. Secondly, the effects of amino acids were examined alone or in combination with insulin. Others have reported the involvement of both lysosomal and ubiquitin-proteasome pathways during incubation of rat skeletal muscle *in vitro *in the presence of 10 mM leucine [24]. This suggests the possibility that the immediate cellular response towards amino acid withdrawal is autophagy either through sequestration of protein substrates to lysosomes or through modification of mRNA levels for lysosomal proteases, but is followed by longer term activation of ubiquitin-proteasome pathway. The activation of lysosomal pathway in response to total amino acid deprivation as reported by the previous study is more likely due to cell stress [13].

*In vitro *studies using L6 or human muscle cells have confirmed the inhibitory effects of insulin on muscle protein degradation [25,26]. Furthermore the mechanism of insulin action appears to be mediated by a decrease in the activity of the ubiquitin-proteasome pathway [25]. Both *in vitro *[9] and *in vivo *[10] studies have demonstrated that insulin down regulates the expression of 14-kDa E2 ubiquitin conjugating enzyme and ubiquitin, respectively. The present study demonstrates that the inhibitory action of insulin on expression of components of the proteasome pathway is decreased when either amino acids or leucine concentrations in the media are decreased to 0.2 × physiological concentrations. *In vivo*, the starvation-induced increase in muscle proteasome-dependent proteolysis is only slowly reversed over 12 h of refeeding rats and acute infusion of amino acids over 3 h fail to alter the expression of components of the proteasome pathway [23]. A 6-hour hyperinsulinaemic, hyper-aminoacidaemic infusion study significantly reduced the mRNA expression for ubiquitin in fast twitch and mixed skeletal muscle [10]. These data combined with our own suggest that under normal fed conditions insulin and amino acids act together to suppress the ubiquitin-proteasome pathway and that the fasting-induced increase in muscle protein degradation is due to lowered insulin and amino acid circulating concentrations resulting in enhanced expression of members of the proteasome pathway.

Numerous studies have demonstrated that insulin and amino acids regulate muscle protein synthesis and hepatic autophagy through the mTOR [18-29]. Inhibition of the mTOR pathway by rapamycin abolishes the effect of leucine media starvation on autophagic proteolysis in C2C12 myotubes [13]. Here, we demonstrate that rapamycin blocked the inhibitory effects of increasing amino acid concentration from 0.2 to 1.0 × PC AA, on protein degradation, but did not block the inhibitory effect of either leucine or insulin. In agreement with the present results, previous studies have demonstrated that leucine suppresses proteolysis in skeletal muscle and hepatocytes by an mTOR independent mechanism[15,30,31]. Since our observations demonstrated that amino acid mixtures suppressed proteolysis in an mTOR dependent manner, this suggests that the mTOR pathway is influenced by amino acids other than the branched chain amino acid group. It has been proposed that glutamate and glutamine have suppressive effects on proteolysis in muscle [32] and studies in other cell types have demonstrated that these amino acids influence the mTOR signalling pathway [33,34]. Phoshorylation targets for mTOR have been proposed to be S6 kinase and 4E-BP1 as well as the phosphorylation dependent control of the degradation of many F-box proteins. Phosphorylation of S6 kinase by insulin greatly depends on amino acid availability [35] and leucine alone has been reported to induce S6 activation [36,37]. We were also able to demonstrate that rapamycin inhibited amino acid or insulin-dependent p70 S6 kinase phosphorylation. These changes were paralleled by a consistent trend for an increase in mRNA for 14-kDa E2 enzyme. The expression of several muscle-specific E3 ubiquitin ligases is consistently increased in conditions causing muscle wasting and mouse knock out studies have demonstrated that these are required for muscle atrophy [38]. Insulin and insulin-like growth factor-1 acting through the PI3-k/AKT pathway, suppress the expression of E3 ligases, MuRF1 and MAFbx/atrogin-1 [39]. Taken together, the present study argues that amino acids act through mTOR to regulate myofibrillar proteasome-dependent proteolysis but further studies are necessary to establish the down stream effector pathways responsible for changes in the expression of components of the proteasome complex.

## Conclusion

In conclusion we provide evidence that depletion of a mixture of amino acid in the media of C2C12 myotubes increases proteasome-dependent-proteolysis and the expression of the components of the ubiquitin-proteasome pathway. Theses effects of amino acids were additive with those of insulin, not simply related to changes in leucine, and appear to be mediated by the mTOR pathway. Further studies are required to establish the exact signalling pathways by which amino acids alter the activity of the proteasome pathway to provide pharmacological targets to combat muscle wasting which is associated with various pathological states, such as prolonged starvation, sepsis, cancer cachexia and diabetes.

## Methods

### Cell culture

Medium, sera, vitamins solution, and antibiotic/antimycotic were bought from GIBCO BRL, Paisley, UK. Proteasome inhibitor MG132 was from Merck Biosciences, Nottingham, UK. Rapamycin was from SIGMA Chemicals Company, Poole, Dorset, UK. The C2C12 mouse cell line was purchased from the ATCC and was used between passage numbers 14 and 19. Cells were grown in Dulbecco's modified Eagle's medium (DMEM) supplemented with 10% (v/v) foetal bovine serum (FBS), 44 mM NaHCO_3_, 25 mM _D_-glucose and 1 mM sodium pyruvate in a humidified environment of 5% CO_2 _and 95% O_2 _at 37°C. Cells were seeded in 12 well plates (for tyrosine release assay) or 6 well plates (for RNA analyses) or in 90 mm dishes (for phosphorylation studies). The cells were grown in the presence of 10% FBS for 2–3 days until confluent, at which time the medium was replaced with fusion medium i.e., DMEM containing 10% horse serum (HS) to induce differentiation. The defined medium used in the studies was made up of vitamin solution, constituents of the RPMI – 1640 Select – Amine™ kit (GIBCO BRL), a mixture of rat physiological concentration amino acids (PC AA) (Table 1), 25 mM _D_-glucose, antibiotic/antimycotic, 24 mM NaHCO_3_, 1 mM Sodium pyruvate, 2 mM tyrosine (specially added to avoid reincorporation of tyrosine in proteolysis measurements) and phenol red. Either 1.0 × PC AA or 0.2 × PC AA media were employed in the studies. The only difference was in the concentration of amino acids. pH of the medium was adjusted to 7.4 with 1 M HCl before use. Where stated MG132 was added at 50 uM and did not have any effect on cell viability.

### Creatine phosphokinase (CPK) and total protein measurement

Cells (passage 16) were seeded in 6-well plates and incubated in 5% CO_2 _and 95% O_2 _at 37°C until confluent. At this stage (Day 0), media was removed from 3 wells, cells were rinsed thrice with ice cold 1 × phosphate buffer saline (PBS) and kept at -80°C in 1 × Hanks balanced salt solution (HBSS; SIGMA) until analyses. Growth medium, in the remaining plates, was replaced with fusion medium and samples in triplicates were then collected every 24 hours for 5 days. CPK activity and protein content in the samples were analysed using commercial kits from SIGMA and BioRad, respectively, according to manufacturer's instructions.

### Tyrosine release assay for measuring proteolysis

Protein degradation was determined by monitoring the release of TCA soluble radioactive tyrosine into the culture medium [40]. Fully differentiated myotubes from day 5 were radiolabelled with 1 μCi _L_[ring -3, 5-^3^H]-tyrosine/ml fusion medium for 48 hours in an atmosphere of 5% CO_2 _and 95% O_2 _at 37°C to label cellular proteins. Monolayers were rinsed with 1 × HBSS and incubated with serum free defined medium for two hours at 37°C to allow the degradation of short live proteins. Myotubes were then rinsed twice with 1 × HBSS and chased once again for 8 hours in defined medium containing 0.05% (w/v) bovine serum albumin (BSA) with or without insulin or other agents. At the end of incubation period media was removed into tubes containing 1 mg BSA and precipitated overnight (for convenience) with an equal volume of 20% (w/v) TCA at 4°C. Tubes were centrifuged at 3000 g at 4°C for 5 min and supernatant containing soluble proteins (A) was separated. TCA insoluble proteins (B) in the pellets and myotubes (C) in the plates were solubilised in 0.5 and 1.0 ml of tissue solubiliser (0.5 M NaOH and 0.1% Triton X-100), respectively. One hundred microlitres of each fraction of protein (A, B and C) was counted using Packard Scintillation counter. Percent protein degradation was calculated by dividing radioactivity in TCA soluble protein (A) by sum of radioactivity in TCA soluble (A), TCA insoluble (B) and in myotubes (C).

### Northern hybridisation for components of ubiquitin-proteasome pathway

Differentiated myotubes were used from day 5. Experimental conditions used for tyrosine release assay were reproduced except that cold tyrosine was used instead of _L_[ring -3, 5-^3^H]-tyrosine. At the end of 8 h incubation period myotubes were rinsed with ice cold 1 × HBS and total RNA was extracted using Tri Reagent (SIGMA) according to the manufacturer's guidelines. The quantity and purity of RNA were assessed by observing absorption at 260 and 280 nm.

Ten micrograms of RNA was run on 1% agarose formaldehyde denaturing gel, transferred to Genescreen™ membrane (NEN Life science products, Inc. Boston, Massachusetts, USA) and covalently fixed to the membrane by UV irradiation. Strippable probes, either of cDNA for 14-kDa E2 ubiquitin conjugating protein, C2 and C8 20 S proteasome subunits and glyceraldehyde 3 phosphate dehydrogenase (GAPDH, a house keeping gene) were synthesized with [α-^32^P] dATP to a specific activity of 10^9 ^cpm/μg cDNA using Ambion Strip EZ DNA kit (AMS Biotechnology ltd, Witney Oxon, UK). Unincorporated radioactivity was removed by passing the synthesized probe through chromaspin – 30 column (Clonetech laboratories ltd, Hampshire, UK). Membranes were prehybridised with Quickhyb hybridisation solution (Stratagene cloning system, California, USA) for 20 min at 65°C. Purified probe containing salmon sperm DNA (Ambion) was denatured at 90°C for 10 min and transferred to the hybridisation solution. Probe was then hybridised to the RNA on the blot for two hours at 65°C. To remove non-specific binding the blot was washed twice with 2 × SSC/0.1% SDS at 60°C for 15 min each and then once with 1× SSC/0.1% SDS at 65°C for 15 min. Autoradiographic signals were quantified by laser densitometry and expressed relative to the corresponding GAPDH mRNA value to normalise for equal loading and transfer of RNA. GAPDH was used because previous studies with nutrients and hormones have documented that mRNA level for it remains constant [10]. Membranes were stripped using Strip EZ kit and rehybridised with other components of the ubiquitin-proteasome pathway as described above.

### Western blotting and immuno detection for p70 S6 kinase phosphorylation

Myotubes from day 6 were incubated over night in 1 × PC AA medium containing 0.05% (w/v) BSA in a humidified atmosphere of 5% CO_2 _and 95% O_2 _at 37°C. Next morning medium was replaced with 5 ml of fresh 1 × PC AA medium containing 0.05% (w/v) BSA and dishes were transferred back to the incubator at 37°C. After 60 min half of the dishes were treated with 2.2 × 10^-8 ^M rapamycin for 20 min at 37°C in 5% CO_2 _and 95% O_2_. Following this half of the rapamycin treated and non-treated dishes were stimulated with 10^-8 ^M insulin for another 20 min. Medium was aspirated and monolayers were rinsed with ice cold 1 × PBS. Cells were lysed by adding pre heated (95°C) buffer composed of 62.5 mM Tris-HCl (pH 6.8), 2% (w/v) SDS, 1.55% (w/v) dithiothretol (DTT), 10% (v/v) glycerol and 0.01% (w/v) bromophenol blue, on them. Cells were scraped of and immediately transferred in to 1.5 ml micro centrifuge tubes and passed through a 25 guage needle 5 times to break cellular nucleoprotein complexes. Cell extracts thus obtained were heated at 95°C for 5 min and stored at -80°C until further analyses.

Equal concentration of protein from cell extracts was resolved on 10% SDS polyacrylamide gel. Gel was run at a constant voltage of 80 for 3 hours and then transferred overnight on to polyvinylidene diflouride (PVDF) membrane (NEN) at a constant current of 300 mA at 4°C. Protein was heat fixed to the membrane at 65°C for 30 min to expose antibody binding epitopes on the antigen. Prior to immunodetection, heat fixed membrane was briefly soaked in 100% methanol, followed by a 5 min wash in 1 × TBS (20 mM Tris-HCl, 137 mM NaCl, pH 7.6) at room temperature. Membrane was blocked in 1 × TBST buffer (1 × TBS containing 0.1% (v/v) Tween-20 and 5% (w/v) non fat dry milk) for 1.5 hour at room temperature. Membrane was incubated overnight with primary antibody, phospho-p70 S6 kinase (Thr 389) (New England Biolabs (UK) ltd., Hertfordshire, England), dilution 1: 1000 in 1 × TBST containing 5% (w/v) BSA at 4°C. The next morning, membrane was washed four times with 1 × TBST for 5 min each at room temperature and incubated with HRP-linked anti-rabbit secondary antibody, dilution 1: 2000 in 1 × TBST containing 5% (w/v) BSA at 25°C for an hour. Finally membrane was washed again as before and HRP-protein substrate complex was detected using lumiglo detection system (New England Biolabs) according to the manufacturer's instructions. Membrane was exposed to Amersham Hyperfilm and the intensity of bands observed was quantified by laser densitometry. The membrane was stripped at 55°C for 20 min in a buffer composed of 62 mM Tris-HCl, pH 8.0, 2% (w/v) SDS, 0.7% (v/v) β mercaptoethanol. The blot was then reprobed for anti-p 70 S6 kinase that can recognise both phospho and dephospho antigenic sites.

### Statistical analyses

Values are presented as means ± SEM. Differences of means were determined by Analysis of variance in a General Linear Model, running Minitab version 13.1. Post hoc comparisons were performed by Tukey's pair wise comparison for multiple treatments. p < 0.05 was accepted as a significant difference between treatments.

## Authors' contributions

FS designed, conducted the experiments & analysed the data and drafted the manuscript. MAL & DGH participated in the design of the studies, data interpretation and preparing the final manuscript. All authors read and approved the final manuscript.

## Figures and Tables

**Table 1:** Concentration of _L_-amino acids in the 1.0 × physiological concentration (PC) culture media.

**Amino acids**	**Concentration (mM)**
Alanine	0.41
Arginine	0.18
Asparagine	0.07
Aspartate	0.04
Cysteine	0.06
Glutamate	0.11
Glutamine	0.74
Glycine	0.28
Histidine	0.07
Isoleucine	0.09
Leucine	0.15
Lysine	0.35
Methionine	0.05
Phenylalanine	0.08
Proline	0.33
Serine	0.53
Threonine	0.25
Tryptophan	0.07
Tyrosine	0.07
Valine	0.19
